# A novel aptamer-G-quadruplex/hemin self-assembling color system: rapid visual diagnosis of invasive fungal infections

**DOI:** 10.1186/s12941-023-00570-6

**Published:** 2023-05-11

**Authors:** Ying Hua, Feng Hu, Xia Ren, Yueling Xiong, Jian Hu, Fan Su, Xiaolei Tang, Yufeng Wen

**Affiliations:** 1 School of Nursing, Wannan Medical College, Wuhu, 241000 Anhui China; 2 Centre of Translational Medicine and Vascular Disease Research Center, The Second Affiliated Hospital of Wannan Medical College, Kangfu Road 10#, Jinghu District, Wuhu, 241000 Anhui China; 3 Department of Blood Transfusion, The First Affiliated Hospital of Wannan Medical College (Yijishan Hospital of Wannan Medical College), Wuhu, 241000 Anhui China; 4 School of Public Health, Wannan Medical College, No.22, Wenchang Xi Road, Wuhu, 241002 Anhui China

**Keywords:** Invasive fungal infections (IFI), Aptamers, Systematic Evolution of Ligands by EXponential enrichment, Self-assembling, Visual diagnosis, G-quadruplex

## Abstract

**Background:**

The clinical symptoms of invasive fungal infections (IFI) are nonspecific, and early clinical diagnosis is challenging, resulting in high mortality rates. This study reports the development of a novel aptamer-G-quadruplex/hemin self-assembling color system (AGSCS) based on (1 → 3)-β-D-glucans’ detection for rapid, specific and visual diagnosis of IFI.

**Methods:**

We screened high affinity and specificity ssDNA aptamers binding to (1 → 3)-β-D-glucans, the main components of cell wall from *Candida albicans* via Systematic Evolution of Ligands by EXponential enrichment. Next, a comparison of diagnostic efficiency of AGSCS and the (1 → 3)-β-D-glucans assay (“G test”) with regard to predicting IFI in 198 clinical serum samples was done.

**Results:**

Water-soluble (1 → 3)-β-D-glucans were successfully isolated from *C. albicans* ATCC 10,231 strain, and these low degree of polymerization glucans (< 1.7 kD) were targeted for aptamer screening with the complementary sequences of G-quadruplex. Six high affinity single stranded DNA aptamers (A1, A2, A3, A4, A5 and A6) were found. The linear detection range for (1 → 3)-β-D-glucans stretched from 1.6 pg/mL to 400 pg/mL on a microplate reader, and the detection limit was 3.125 pg/mL using naked eye observation. Using a microplate reader, the sensitivity and specificity of AGSCS for the diagnosis of IFI were 92.68% and 89.65%, respectively, which was higher than that of the G test.

**Conclusion:**

This newly developed visual diagnostic method for detecting IFI showed promising results and is expected to be developed as a point-of-care testing kit to enable quick and cost effective diagnosis of IFI in the future.

**Supplementary Information:**

The online version contains supplementary material available at 10.1186/s12941-023-00570-6.

## Introduction

Invasive fungal infections (IFI) result from opportunistic fungal pathogens that mainly invade the bloodstream including internal organs of usually immunocompromised individuals [1]. Primarily, IFI are caused by fungal species that include *Candida* spp., *Aspergillus* spp., *Mucorales* spp., *Cryptococcus* spp., and *Pneumocystis* spp. [1–3]. In China, most IFI are attributed to *Candida albicans* [4]. The incidence of IFI is increasing among immunocompromised individuals including subjects of extensive chemotherapy, treatment with broad-spectrum antibiotics, and corticosteroids. Vulnerability to IFI has also been reported among people who have diabetes mellitus, skin burns, HIV infection, neutropenia and catheter [5]. Currently, the challenging late diagnosis of IFI has contributed to high IFI-associated mortality rates [5, 6]. Therefore, early diagnosis is critical to provide the best treatment strategy and improve the prognosis for IFI patients.

**Fig. 1:**
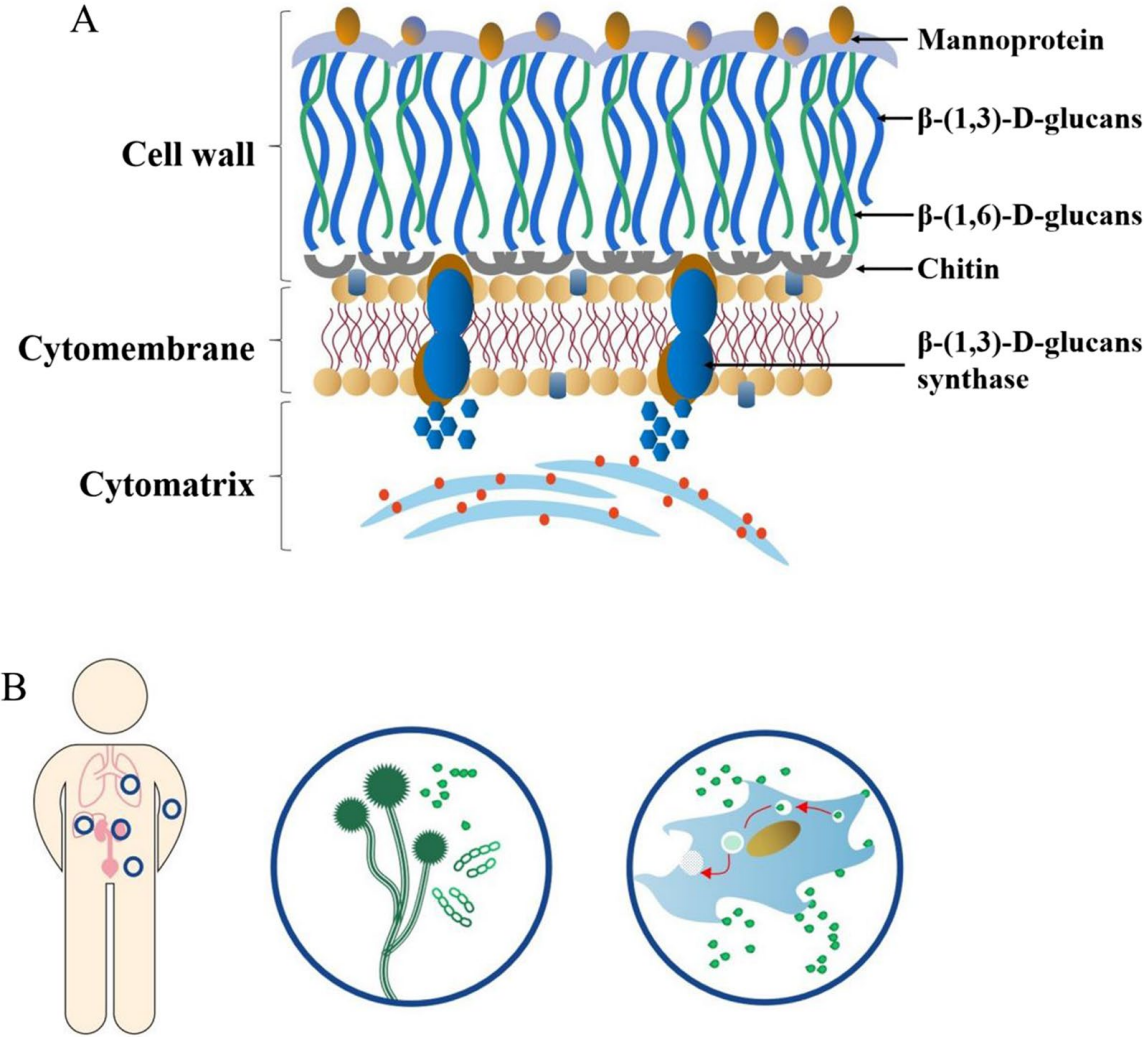
Cell structure and invasion mode of *Candida albicans.* **A** The cell wall of *Candida albicans* consists of an endoskeletal layer containing chitin and β-glucan ((1 → 3) -β-D-glucan and (1 → 6) -β-D-glucan) and an outer layer containing highly glycosylated mannoprotein, of which approximately 84% is water insoluble (1 → 3) -β-D-glucan. The mannan portion of the cell wall accounts for 40% of its dry weight, and the core structure of N-mannan is a focused dihydrochlorol phosphate oligosaccharide consisting of 3 glucose, 9 mannose and 2 N-acetylglucosamine residues. The outer branched mannan is linked to the N-mannan nucleus via the α-1, 6-backbone. The cell membrane is composed of phospholipid bilayer and β-(1,3)-D-glucans synthase. **B** *Candida albicans* completes the invasion process through two mechanisms: one is endocytosis induced by host cells, and the other is osmosis activated by *Candida albicans* hyphae

The cell wall of *Candida albicans* consists of an inner skeletal layer containing chitin and β-glucan ( (1 → 3) -β-D-glucan and (1 → 6) -β-D-glucan) and an outer layer containing highly glycosylated mannoprotein, of which about 84% is water insoluble (1 → 3) -β-D-glucan (Fig. 1A). Yeast cells adhere to host cell surfaces by the expression of adhesins. Contact to host cells triggers the yeast-to-hypha transition and directed growth via thigmotropism. The expression of invasins mediates uptake of the fungus by the host cell through induced endocytosis. Adhesion, physical forces and secretion of fungal hydrolases has been proposed to facilitate the second mechanism of invasion, i.e., fungal-driven active penetration into host cells by breaking down barriers (Fig. 1B). In clinical practice, the diagnosis of IFI is currently based on three rudimental methods such as: (i) clinical examination, (ii) radiological tests and (iii) mycological tests. Among these methods, serological tests are used clinically, and the common methods are: Aspergillus galactomannan antigen test (GM test) and 1, 3-β-D-glucan antigen test (G test). These two methods have been approved for use in Europe, America and other countries, but each has certain limitations. For example, G test only targets Aspergillus infection, and is ineffective for other fungi detection, and the sensitivity and specificity are affected by many factors. Although G test can detect more pathogenic fungi including Aspergillus and Candida, and preliminary clinical studies show good sensitivity and specificity, it cannot detect zygomycetes and cryptococcus, nor can it identify specific bacterial genera and species. Radiological examination mainly uses lung high resolution CT and bronchoscopy to help confirm the diagnosis. The mycological examination involves fungal culture, DNA-based tests, and antigen detection. Although a fungal culture can identify the fungal species causing an infection and further inform treatment strategies by *in-vitro* antifungal susceptibility testing, culture methods have disadvantages of low success rate, and they are time-consuming [7]. Molecular based methods such as polymerase chain reaction (PCR)- have significantly higher sensitivity and rapid detection compared to fungal cultures. However, such methods are associated with high costs and require specialized technical equipment [7]. Antigen detection tests (especially (1 → 3)-β-D-glucans assay) have high sensitivity but require high-quality samples and may yield false-positive results when samples are taken from patients receiving immunoglobulin, albumin, coagulation factors, or other blood products [8]. In addition, Tang et al. [9] used SELEX to screen out aptamers that specifically recognize (1 3) -β-D-glucans. This method has achieved good results, but it can still be further optimized. Taken together, because of the highlighted disadvantages of traditional diagnostic methods for IFI it is therefore justified to explore a neotype detection method that could be highly sensitive, specific, rapid and accurate.

Except for being the blueprint of life or bearing biological information, single-stranded nucleic acids present important catalytic functions including ligand binding because of their ability to fold into specific conformations. Nucleic acids involved in such processes are referred to as functional nucleic acids (FNAs) [9]. In this study, two types of FNAs were used. One of these FNAs is an aptamer for biorecognition, which comprises synthetic, single-stranded DNA (ssDNA) or RNA molecules that can fold into specific 3D structures that target molecules (e.g., cells, proteins with different molecular weights, or organic and inorganic compounds) and bind to specificity with high affinity [10–14]. They have significant advantages over antibodies, such as low toxicity and immunogenicity, low cost of production, high reproducibility, chemical stability, high target specificity, sensitivity, and can be modified easily [15–17]. Nucleic acid ligands (aptamers), have been widely used for the development of sensors and biomedical applications. The other type of FNA is the horseradish peroxidase (HRP)-mimicking nucleases, which forms a four-stranded structure with G-rich oligonucleotides [18, 19]. As a ligand, hemin can bind to several parallel G-quadruplexes with high affinity and specificity. The G-quadruplex/hemin complex can catalyze hydrogen peroxide (H_2_O_2_) to generate large numbers of reactive oxygen species, which can efficiently catalyze the tetramethylbenzidine (TMB)-H_2_O_2_ system for signal amplification [20]. Due to these advantages, the G-quadruplex/hemin system based on aptamers have been broadly used for detection of ochratoxin A, immunoglobulin E, aflatoxin B1, and cytokines [21–24].

In most fungal cell walls, about 84% of fungal cell wall is composed of (1 → 3)-β-D-glucans [25, 26]. The levels of (1,3)-β-D-glucan in the blood can act as bio-marker for IFI. Therefore, in this study, nucleic acid aptamers with high and specific binding affinity to (1 → 3)-β-D-glucans were screened using the *in-vitro* selection process (SELEX) [27]. The high affinity aptamers selected for bio-recognition were used to develop a new aptamer-G-quadruplex/hemin self-assembling color system (AGSCS) (G-quadruplex DNAzyme for a catalytic unit) to achieve economical, time-efficient, convenient, and accurate diagnosis of IFI.

## Materials and methods

### Reagents

Restriction enzymes Xba I, Hind III and T4 ligase were purchased from Fermentas (Thermo Fisher Scientific, Germany). DNA markers and protein markers were purchased from LabLead Co., Ltd (Beijing, China). PCR mix was purchased from JiErDun Co., Ltd (Shanghai, China). Salmon sperm DNA, hemin, and TMB were purchased from TaiTianHe Co., Ltd (Jinan, China). A turbidimetric (1,3)-β-D-glucan test kit was purchased from Bokang Marine Biological Company (Zhanjiang, China). Barley glucan, curdlan, mannan, endotoxin, dextran, and (1,3)-β-D-glucanase were sourced from Sigma–Aldrich (St. Louis, MO). ssDNA libraries and primers were synthesized by Sangon Biotech Co., Ltd (Shanghai China).

### Extraction of (1 → 3)-β-D-glucans

The strain (*C. albicans* ATCC 10,231) was purchased from the China Center for Type Culture Collection affiliated with Wuhan University. This strain was cultured on Sabaurauds agar (SAB) medium and incubated (48 h at 34 °C).

The (1 → 3)-β-D-glucans were isolated from *C. albicans,* as previously described [29]. The extraction was done as follows, lane 1: The extraction of rude (1 → 3)-β-D-glucans from Candida albicans with Chemical method and ultrasonic crushing; lane 2:Deionized water blank control; lane 3 to lane 6: The rude glucans was digested with (1,3)-β-D-glucanase in different times,1 h, 1.5 h, 2 h and 2.5 h respectively. After treatment, Candida albicans (1,3)-β-dextran, analyzed with silver nitrate staining and periodic acid sif staining, respectively. It was then digested with (1 → 3)-β-D-glucans (Sigma Aldrich) to give a less polymerized water-soluble (1 → 3)-β-D-glucans (< 1.7 KD) as the target of aptamer screening. Low degree polymerization (1 → 3)-β-D-glucans were separated using 10% sodium dodecyl sulfate–polyacrylamide gel electrophoresis (SDS-PAGE) and identified via periodic acid–Schiff (PAS) staining. Next, The purified low degree of polymerization (1,3)-β-d-dextran was analyzed by high performance liquid chromatography and elemental analysis.

### *In-vitro* identification of anti-(1 → 3)-β-D-glucans aptamers

Aptamers with high and specific binding affinity to (1 → 3)-β-D-glucans from *C. albicans* were screened, as previously described [28, 29]. A random ssDNA aptamer library (10^14^ ~ 10^15^) was synthesized, containing 50 random nucleotides (50N), and flanked by fixed sequences (the complementary fragments of the G-quadruplex): 5′-TCTAGA*ATCCCAATCCCAATCCCA*-50N-*ACCCTA*AAGCTT-3′ (86 nucleotides, about 3.4KD; “N” denotes any of the bases A, T, G, and C) was used as the initial pool. The underlined fragments refer to the cutting sites of the restriction enzymes *Xba* I and *Hind* III, respectively. The italicized fragments correspond to the reverse sequences of the G-quadruplex. Primer 1 (P1: 5′-TCTAGAATCCCAATCCCAATCCCA-3′) and primer 2 (P2: 5′-AAGCTTTAGGGT-3′) were used for PCR-based amplification of the DNA library.

The selection steps were as follows: first, 50 μL of purified (1,3)-β-D-glucan (10 μg/mL) were coated on polystyrene microwells with carbonate buffer (pH = 9.6) for 1 h at 37 °C; subsequently, wells were blocked at ambient temperature (25 °C) for 1 h using salmon sperm DNA. Before adding to the coated wells, the initial ssDNA library was suspended in screening buffer (136.9 mM NaCl, 3.98 mM MgSO_4_, 2.68 mM KCl, 1.8 mM CaCl_2_, 1.47 mM KH_2_PO_4_, and 8.06 mM Na_2_HPO_4_), heated (10 min at 95 °C), and instantly placed on ice (5 min). Subsequently, 1 nmol initial ssDNA library was added to the coated wells and incubated (1 h) in a water bath (37 °C). The liquid in wells was discarded and the wells were washed with buffer (screening buffer containing 0.1% Tween-20) three times. Thereafter, 100 μL of deionized H_2_O was added into coated wells, and the wells were heated (95 °C for 10 min). The eluted ssDNA was removed from the wells and used as the template for asymmetric PCR amplification for next screening round. These PCR reactions were performed in 50 μL total volumes, containing 2 μL of template DNA, 25 μL of PCR mixture (2×), 1 μL of each primer (10 M), and 24 μL of nucleic acid-free distilled water on a Bio-Rad PCR System (T100, Bio-Rad, USA). PCR conditions included a pre-warming step of 5 min at 95 °C, 12 cycles performed; 95 °C for 30 s, 60 °C for 30 s, and 72 °C for 20 s, a final extension step at 72 °C for 10 min followed. The complete *in-vitro* selection required a total of eight selection cycles.

Finally, we selected the highest affinity pool among the eight pools and amplified the ssDNA of the highest affinity pool to yield double-stranded DNA (dsDNA) by routine PCR. The dsDNA and the pUC19 plasmid were cut by Xba I and Hind III and ligated using a T4 ligase. The recombinant plasmids were transferred into *E. coli* DH5α, and 40 single aptamer clones were randomly picked and amplified by PCR for identification. Plasmids from 40 positive clones were PCR-amplified with a biotin-labeled primer for generation of biotinylated single ssDNA aptamers for subsequent evaluation of their affinity to (1 → 3)-β-D-glucans by enzyme-linked oligonucleotide assay (ELONA).

### Binding affinity and specificity of selected individual aptamers determined by ELONA

For testing the binding affinity, 96-well plates were treated with 50 μL of purified (1,3)-β-D-glucan (10 μg/mL) and incubated overnight (4 °C), washed thrice with phosphate-buffered saline (PBS), then blocked for 1 h at 37 °C with salmon sperm DNA (100 μL, 10 μg/mL). The different types of (1 → 3)-β-D-glucans (curdlan and barley glucan) and non-(1 → 3)-β-D-glucans (mannan, endotoxin, and dextran) were solubilized according to the suppliers’ instructions. These polysaccharides (10 μg/mL) were coated on 96-well plates and left at 4 °C overnight, rinsed three times with PBS, and blocked with salmon sperm DNA (100 μL, 10 μg/mL; 1 h at 37 °C).

Biotin-labeled aptamers (10 nM) were then added to the above-described microwells coated with different substances and incubated (1 h at 37 °C). Post rinsing the wells with PBS (five times) to remove excess unbound aptamers, HRP-conjugated streptavidin was added and left to incubate (37 °C for 30 min). Subsequently, the wells were washed three times using PBS. Finally, TMB substrate and stop buffer (2 M H_2_SO_4_) were added and absorbance values were measured at optical density (OD)_450_ nm using a microplate reader.

### Development of the aptamer G-quadruplex/hemin self-assembling color system (AGSCS)

**Scheme 1.**
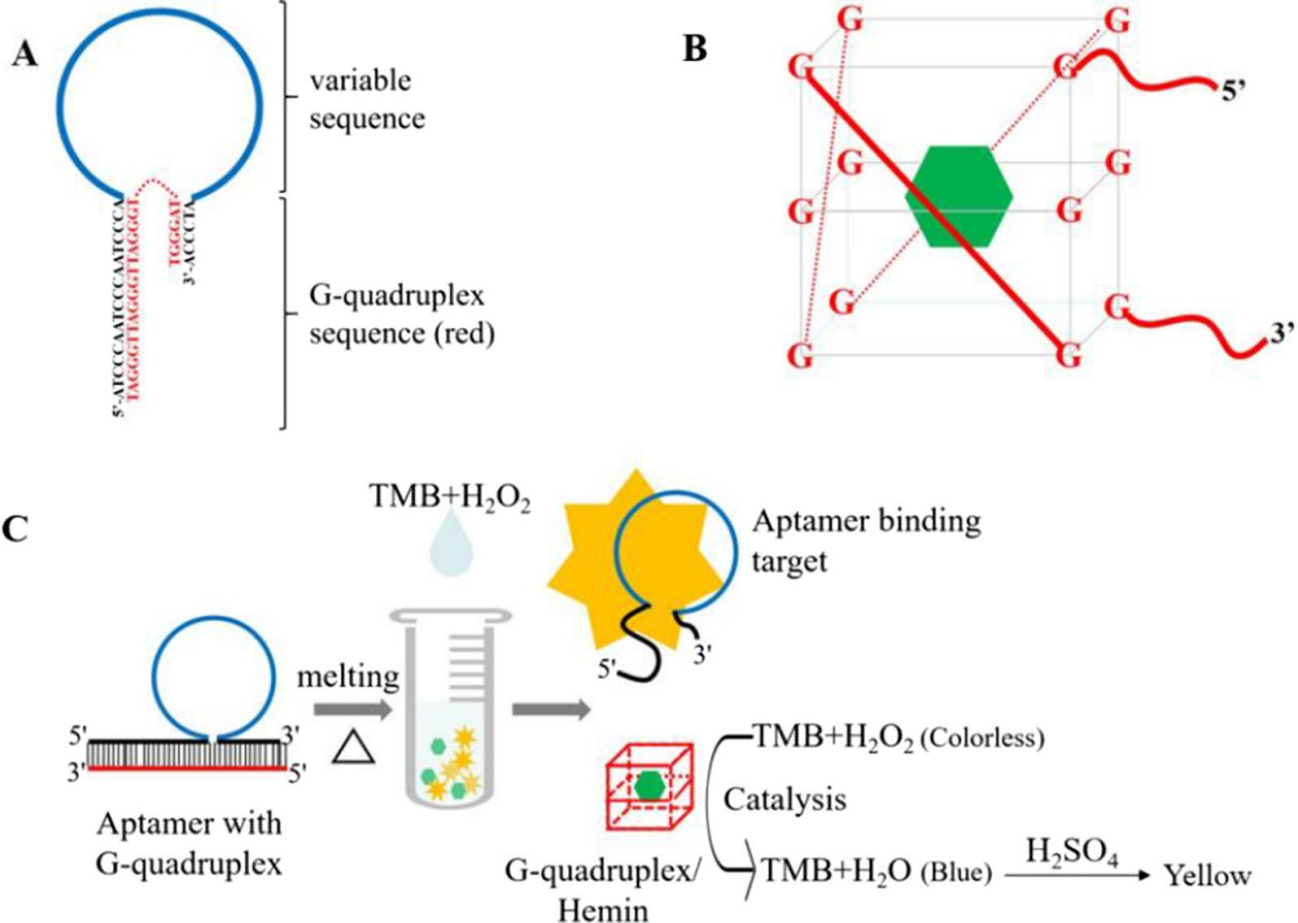
Schematic diagram of the elements and working process of the aptamer G-quadruplex/hemin self-assembling color system (AGSCS).** A** Diagram of the structural composition of the aptamer with G-quadruplex. The blue non-closed ring represents the aptamer; the black bases reflect the complementary sequences of the G-quadruplex; the red bases represent the G-quadruplex sequences. **B** Diagram of the structural composition of the G-quadruplex/hemin complex. The “cube” comprises 12 guanines from the same G-quadruplex line, with every four guanines distributed in a plane. The green hexagon represents hemin. **C** The working process of the AGSCS. The aptamers with G-quadruplex were added to the samples and mixed carefully. In the presence of (1 → 3)-β-D-glucans, aptamers bound the target tightly, the hairpin structure of the aptamer probe was opened, and the G-quadruplex was released. The free G-quadruplex was folded into a cube containing hemin with the aid of hemin in the system. The G-quadruplex/hemin complex showed peroxidase-catalytic activity and catalyzed the tetramethylbenzidine/H_2_O_2_ system to change color, from colorless to blue. After the addition of stop solution, there was a color change from blue to yellow, and the color could be detected at 450 nm by a microplate reader or the naked eye

**Scheme 2:**
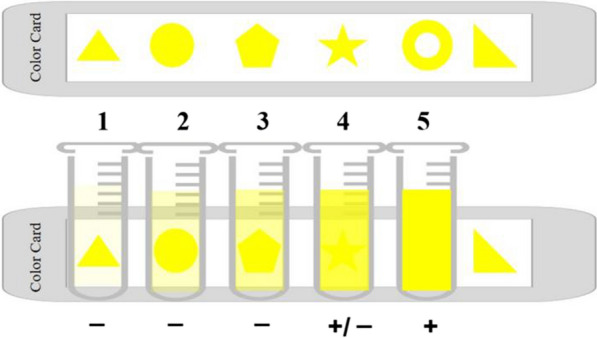
Schematic diagram of results scored by the naked eye with color card. **A** To score the results via naked eye, a color card was designed as a tool for enabling accurate and objective diagnosis. In this card, six different patterns in the same color, were distributed in a row as indicated in the diagram. **B **The pattern was covered by tubes containing different shades yellow, from tube 1 to tube 5. If the pattern could be distinguished by the naked eye [e.g., as is the case with tubes 1, 2, and 3 in **B**, the tube containing the sample should be scored as negative, and the patient would not be categorized as having invasive fungal infections (IFI). If no pattern was observed (e.g., as is the case with tube 5), the tube containing the sample should be scored as positive, and the patient would be considered to have IFI. If the pattern could be distinguished faintly (e.g., tube 4), the tube containing sample should be scored as weakly positive, and the patient could potentially have IFI

Equal amounts of aptamers (a mixture of four aptamers [A1, A4, A5, and A6]) and G-quadruplex were mixed to combine G-quadruplexes with their complementary sequences in the tail of the aptamers (Scheme 1a). In the presence of (1 → 3)-β-D-glucans, aptamers specifically recognized and bound to (1 → 3)-β-D-glucans, the hairpin structure of the aptamer probe was opened, and the G-quadruplex was released, resulting in the formation of a G-quadruplex/hemin complex (Scheme 1b) with the aid of hemin, which exhibited biocatalytic abilities in the process of H_2_O_2_ electroreduction and generating an amplification signal for (1,3)-β-D-glucan detection. However, in the absence of (1 → 3)-β-D-glucans, the G-quadruplex binds to complementary sequences in the aptamer tail to form a short double-stranded DNA (dsDNA). Hence, the G-quadruplex would not be released from the aptamer, thus the G-quadruplex/hemin would not be formed in presence of hemin, and the reaction with Scheme 1 and 2 TMB and H_2_O_2_ to generate a visible color change for (1 → 3)-β-D-glucans detection would not be catalyzed (Scheme 1c).

### Quantitative detection of (1,3)-β-D-glucan by the AGSCS

Different concentrations of (1,3)-β-D-glucan from 0 to 400 pg/mL (400 pg/mL, 200 pg/mL, 100 pg/mL, 50 pg/mL, 25 pg/mL, 12.5 pg/mL, 6.25 pg/mL, 3.125 pg/mL, 1.6 pg/mL, 0.8 pg/mL, and 0 pg/mL) were used to coat 96-well plates (50 μL/well), which were kept overnight (4 °C), washed with PBS (three times), and blocked with salmon sperm DNA (100 μL, 10 μg/mL) for 1 h at 37 °C. Subsequently, 100-nM aptamers (a mixture of four aptamers [A1, A4, A5, and A6]) and 100-nM G-quadruplex were mixed to make G-quadruplexes combine with their complementary sequences in the tail of aptamers. Aptamers with G-quadruplex were added to the above-mentioned wells, and hemin was added at the same time. Plates were incubated (1 h at 37 °C) to form the AGSCS. After adding TMB/H_2_O_2_ mixture substrate for coloration and 2 M H_2_SO_4_ stop buffer, absorbance values were measured at OD_450_ nm using a microplate reader.

### Participants

Serum samples from 82 patients with IFI (including 14 patients with a central venous catheter, eight patients subject to thoracic surgery, 10 patients with severe burns, 27 patients with cancer, and 23 patients with chronic respiratory failure) were taken from Second People’s Hospital of Wuhan, Zoucheng People’s Hospital, and the Second Affiliated Hospital of Wannan Medical College from February 2016 to February 2019. The patients had an average age of 56.2 years, including 45 males and 37 females. The inclusion criteria were as follows: (1) positive fungal culture or body fluid smear; (2) symptoms and signs of fungal infection (e.g., hypotension and/or fever); (3) documented curative effects of anti-fungal therapy; and (4) positive G test and procalcitonin test. All studied participants were required to meet at least three of these criteria. Serum samples from 116 healthy individuals were collected from the Physical Examination Center of the Second Affiliated Hospital of Wannan Medical College and the Second People’s Hospital of Wuhu. The ethics committee of Second Affiliated Hospital of Wannan and other three hospitals mentioned above approved the study. All participants gave written informed consent.

### (1,3)-β-D-glucan detection in human serum samples using AGSCS

The AGSCS procedure was carried out as follows: at first, a 96-well enzyme-linked immunosorbent assay (ELISA) plate was irradiated with ultraviolet light (2 h) and blocked using salmon sperm DNA (100 μL, 10 μg/mL; 1 h at 37 °C). A 50 μL volume serum sample, was added to the wells, and at the same time, aptamers with G-quadruplex and hemin were added, and plates were incubated (37 °C for 1 h). Finally, a TMB substrate together with the stop buffer were added to the wells. A microplate reader was used to measure absorbance at OD_450_ nm. Given our research purpose to develop a simple test, the AGSCS test results were also read by the naked eye. A simple and objective criterion was introduced for scoring the samples as “negative,” “positive,” or “slightly positive,” as indicated and explained in Scheme 2.

### G test (commercial)

Quantification of (1,3)-β-D-glucan in 198 clinical serum samples was done using a turbidimetric (1,3)-β-D-glucan test kit (Cat#KT-120, Zhanjiang A & C biological Ltd. China) following the manufacturer’s protocol. For positive sample classification, the concentration of (1,3)-β-D-glucan needed to be ≥ 100 pg/mL. All positive samples were subject to repeated testing and considered positive only if the repeat result was also positive.

### Guinea pig model

Male guinea pigs (weighing approximately 0.5 kg) were purchased from the Laboratory Animal Center of Wuhan University (Wuhan, China). Ten guinea pigs were injected subcutaneously with triamcinolone acetonide (Sigma-Aldrich) at 300 mg/kg body weight and immunosuppressed twice daily. On day 4, Candida albicans (5 107 CFU) is given intravenously in the saphenous vein, followed by triamcinolone acetonide reduced to 150 mg/kg once daily. Serum (1 3)-β-d-glucan levels are measured every 2 days with G-test and AGSCS.

### Statistical analysis

Quantitative data were reported as mean ± standard deviations (SD) from three replications. Data analysis was done using IBM SPSS (version 25.0 for Windows (IBM, Armonk, NY, USA). One-way ANOVA with Student’s *t*-test was used to compare means. The chi-square test was used to compute the deviations between categorical variables. The probability, *P* < 0.05 were classified as statistically significant.

## Results

### Extraction, isolation, and identification of (1 → 3)-β-D-glucans

**Fig. 2:**
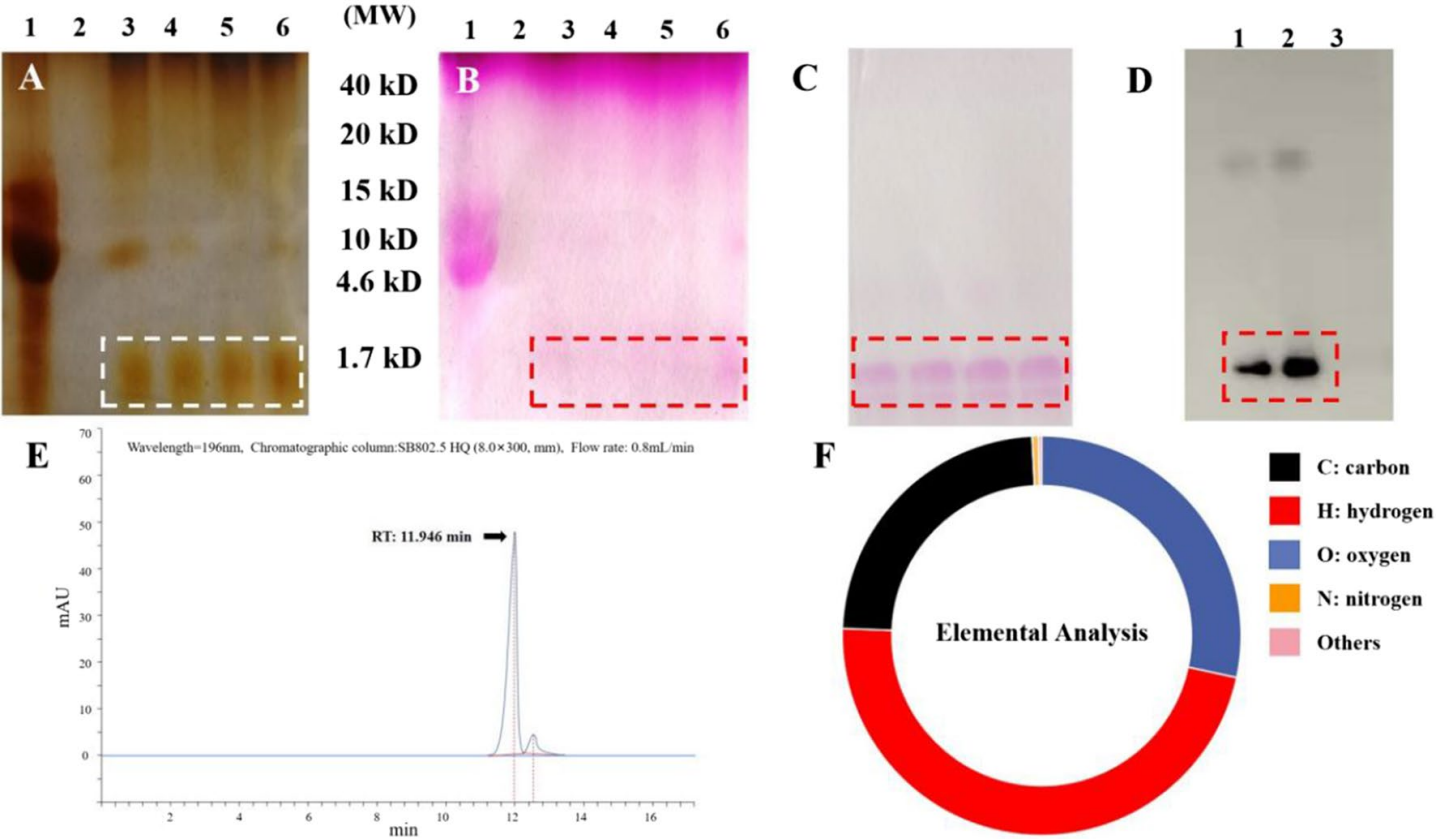
Separation and identification of (1,3)-β-D-glucans from *Candida albicans.* **A** Silver nitrate stainingand, and **B** Periodic acid-schiff staining: The (1 → 3) -β-D-glucan from Candida albicans was treated and analysed using Silver nitrate staining and periodic acid-schiff staining respectively; lane 1: The extraction of rude (1 → 3) -β-D-glucan from *Candida albicans* with Chemical method and ultrasonic crushing; lane 2:Deionized water blank control; lane 3 to lane 6: The rude glucans was digested with (1 → 3) -β-D-glucan in different times,1 h, 1.5 h, 2 h and 2.5 h respectively. **C** The low polymerization degree of (1 → 3) -β-D-glucan was purified by molecular sieve and stained by PAS; Dotted box indecated low molecular weight (1 → 3) -β-D-glucan as target in; **D** The interaction between aptamer and glucan was evaluated by western blot. Lane 1: 1 mg /ml; lane 2: 3 mg /ml; lane 3: glucose monomer, as control; **E** and **F** The purified low polymerization degree of (1 → 3) -β-D-glucan was anlysed by HPLC and elemental analysis. Arrow indicated the retention time of targeted product in HPLC system

Water-soluble (1 → 3)-β-D-glucans were obtained through the extraction and digestion of *C. albicans* ATCC 10,231 strain. The use of *C. albicans* in this study was consistent with that previously used (Tang et al.,2016)[29]. Digested dextrans isolated with SDS-PAGE, identified by silver nitrate staining (Fig. 2A) and (Fig. 2B) PAS staining, molecular weight shown at 1.7 KD. Figure 2A, B and C are cropped from the original (Additional files 4, 5 and 6). (1 → 3)-β-dextran with low polymerization degree purified with molecular sieve, PAS staining; The dotted box indicates a low molecular weight (1 → 3)-β-d-dextran as the target (Additional file 4 and Fig. 2C); The interaction between aptamer and glucan was evaluated by western blot. After binding with aptamer (G tetramer) on PVDF membrane, luminous substrate was added for development (Fig. 2D). Next, we extracted water-soluble (1 → 3)-β-dextran (< 1.7 kD) from agarose gels. Products with low polymerization of purified (1 → 3)-β-D-dextran were analyzed by high performance liquid chromatography and elemental analysis. The target product stays in the HPLC system for 11.946 min (Fig. 2E). Elemental analysis showed that C:H:O was 1:2:1 (Table 1), which is consistent with the elemental composition characteristics of carbohydrates (Fig. 2F). The above results indicate that the isolated (1 → 3)-β-D-glucans were purified carbohydrates.

**Table 1:** Analysis of four elements from low polymeric degree water-soluble (1,3)-β-D-glucans

Element	C	H	O	N
Relative proportion	1.012	2.034	1.068	0.027

### Screening and identification of aptamers with high binding affinity to (1 → 3)-β-D-glucans

**Fig. 3:**
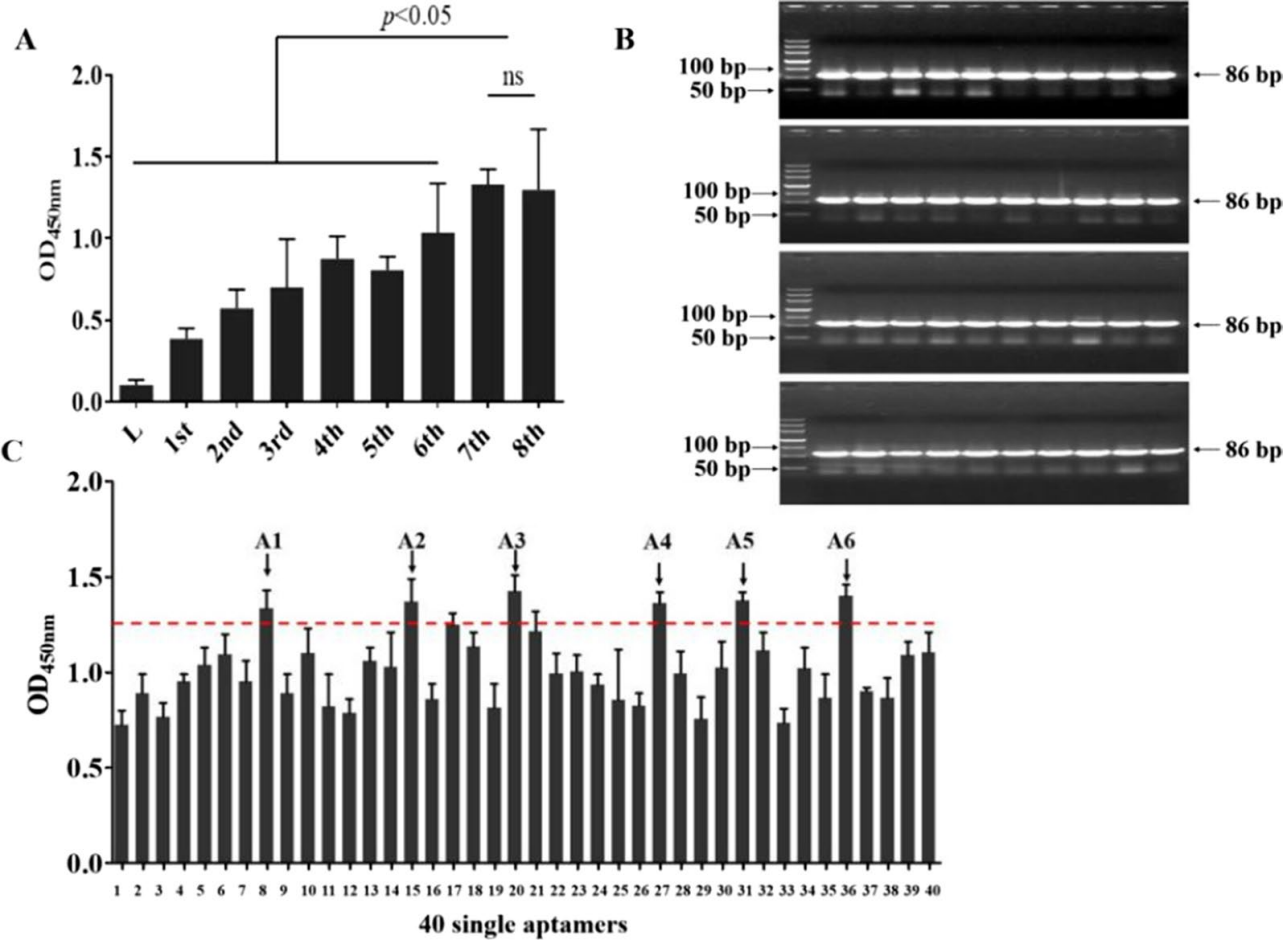
Relative aptamer binding force against the low degree polymeric (1 → 3)-β-D-glucans. **A** The enzyme-linked oligonucleotide assay (ELONA) was used to analyze the relative binding force of the different single-stranded DNA (ssDNA) pools of the (1 → 3)-β-D-glucans. L stands for initial ssDNA library. **B** The 40 random-selected *E. coli* DH5α strains containing pUC19-aptamers were amplified to yield double-stranded DNA aptamers by PCR and identified by agarose gel. **C** ELONA was used to analyze the relative binding strength of the 40 randomly picked single aptamers from the 8th round of collection to the (1 → 3)-β-D-glucans. All data are shown as the means ± standard deviation, and experiments were performed in triplicates. The aptamers with topmost relative affinity are indicated by arrows ( “A1” ~ “A6” from left to right)

The created ssDNA library was deposited into polystyrene micro-wells treated with (1 → 3)-β-D-glucans for aptamer screening. Any unbound ssDNA was discarded, and the bound ssDNA was collected and amplified by asymmetric PCR for the next screening. With each successive screening round, the binding affinity of the aptamer pool steadily improved until the 4th round. However, a minor decline in binding affinity was observed for the 5th screening round. Subsequently, as the number of screening rounds increased, the binding affinity of the aptamer pool progressively increased, and it was higher than the previous 4th screening round until the 7th screening round, reaching stagnation at the 8th screening round (Fig. 3A). As shown in Fig. 3A, the aptamers of the 8th pool had higher affinity to (1 → 3)-β-D-glucans than other pools. A pUC19 cassette was used as a vector to clone the high affinity single aptamers from the 8th pool, and 40 single aptamer positive colonies (Fig. 3B) were picked for evaluation of binding affinity. The single aptamer designated A1–A6 exhibited the topmost binding affinity to the (1 → 3)-β-D-glucans (Fig. 3C).

### Specific binding of aptamers to (1 → 3)-β-D-glucans

**Fig. 4:**
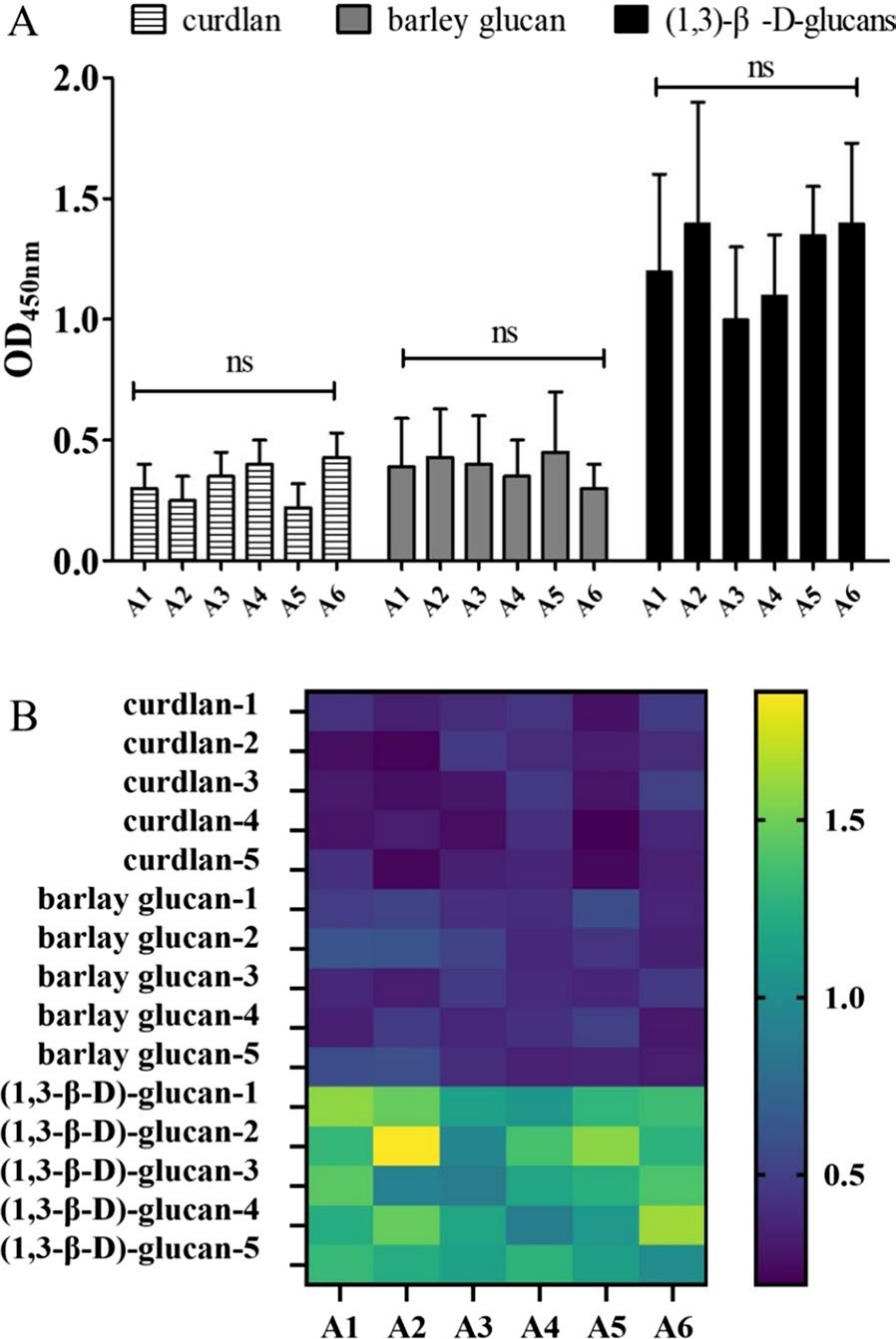
Binding specificity of selected single aptamers, A1–A6, to (1 → 3)-β-D-glucans (curdlan, barley glucan, and (1 → 3)-β-D-glucans obtained from *Candida albicans*) and non-(1 → 3)-β-D-glucans (mannan, endotoxin, and dextran). **A** The binding specificities of selected single aptamers (A1 ~ A6) to (1 → 3)-β-D-glucans were evaluated by enzyme-linked oligonucleotide assay. The absorbance was proportional to affinity between corresponding aptamer-polysaccharide pair, and vice versa. **B** We used heat maps to describe the binding specificity of the single aptamer A1 A6 to (1 3) -β-D-glucan and non-(1 3) -β-D-glucan. All data were expressed as mean standard deviation, and the experiment was repeated five times

To detect binding specificity of the aptamers A1–A6 were challenged with diverse types of (1 → 3)-β-D-glucans (curdlan, barley glucan, and (1 → 3)-β-D-glucans extricated from *C. albicans*) and non-(1 → 3)-β-D-glucans (mannan, endotoxin, and dextran). The selection of these polysaccharides was based on previously published work that reported on the specificity of the glucan-specific ELISA and G test [30, 31]. We observed that A1–A6 displayed a higher affinity for (1 → 3)-β-D-glucans than for non-(1 → 3)-β-D-glucans (*P* < 0.001, *t*-test; Fig. 4A, B), which suggested that A1–A6 could specifically recognize (1,3)-β-D-glucan targets.

### Binding domain of selected aptamers to (1 → 3)-β-D-glucans

**Fig. 5:**
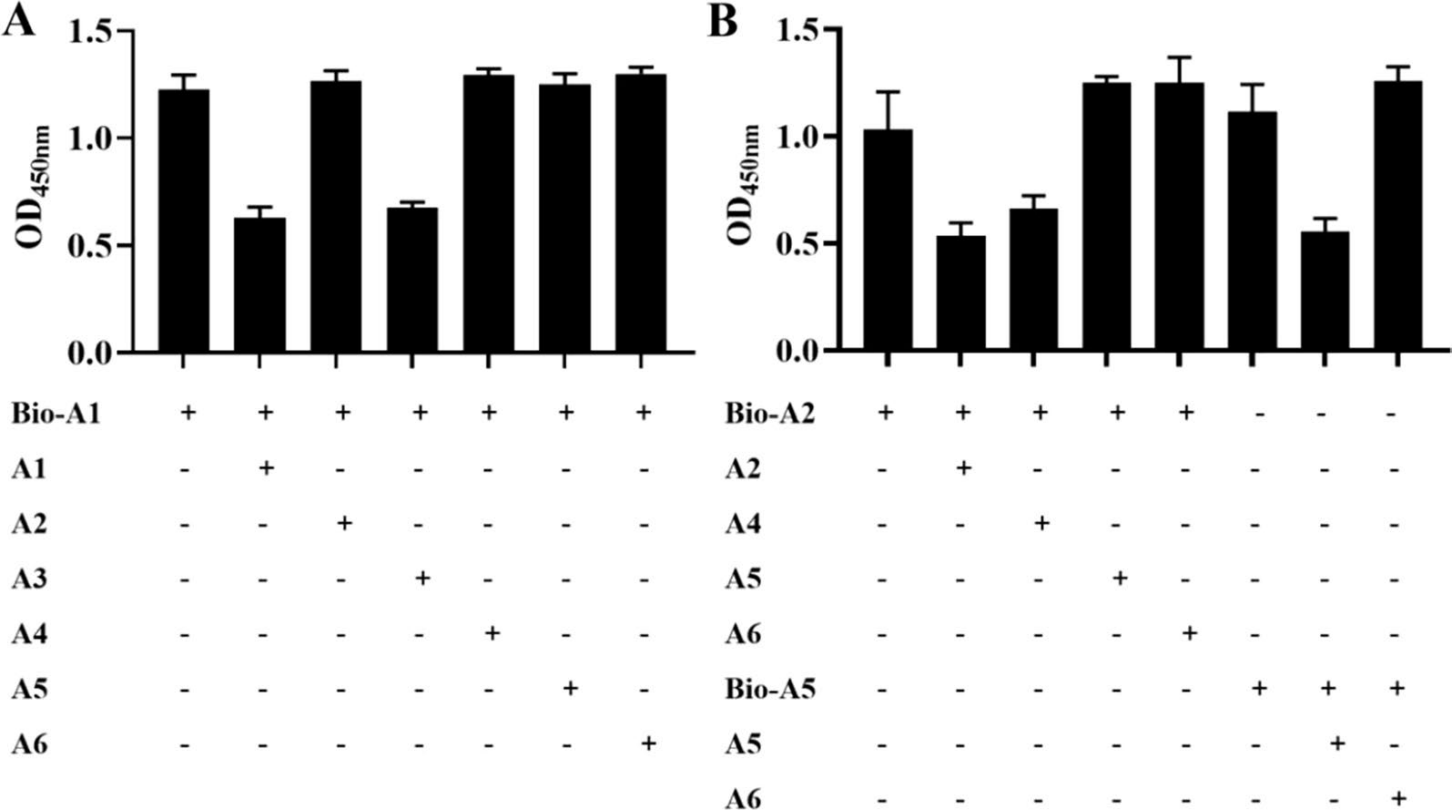
Binding domain of selected aptamers (A1 ~ A6) to (1 → 3)-β-D-glucans.** A** and** B** Biotin-labeled aptamers and unlabeled aptamers were simultaneously added to 96-well plates coated with (1 → 3)-β-D-glucans, and the binding domain of selected aptamers to (1 → 3)-β-D-glucans was confirmed by competitive enzyme-linked oligonucleotide assay. All data shown were computed as the mean ± standard deviation, with all experiments having been performed in triplicates

Biotin-labeled and non-labeled aptamers were added simultaneously to 96-well plates coated with (1 → 3)-β-D-glucans, and the binding domain of selected aptamers to (1 → 3)-β-D-glucans was determined by ELONA. The binding affinity between biotin-labeled A1 (Bio-A1) and (1 → 3)-β-D-glucans was uninfluenced by the presence of A2, A4, A5, and A6; however, it was dramatically decreased in the presence of A3 (Fig. 5A.). As presented in Fig. 5B, the binding affinity between Bio-A2 and (1 → 3)-β-D-glucans was not influenced by the presence of A5 and A6; however, it was evidently declined in A4 presence. In presence of A6, the binding affinity between Bio-A5 and (1 → 3)-β-D-glucans was unimpacted (Fig. 5B). After secondary structure prediction using mfold software (Additional file 1: Fig. S1, Additional file 2: Fig. S2, Additional file 3: Fig. S3 and Additional file 7). These results demonstrate that A1 and A3 had a common binding domain of (1 → 3)-β-D-glucans, A2 and A4 had a common binding domain of (1 → 3)-β-D-glucans, and A5 and A6 had altered binding domains of (1 → 3)-β-D-glucans. As a result, A1, A4, A5, and A6 aptamers were chosen for the investigation of detection performance in the following experiment.

### Detection performance of AGSCS to varying (1 → 3)-β-D-glucans concentrations

**Fig. 6:**
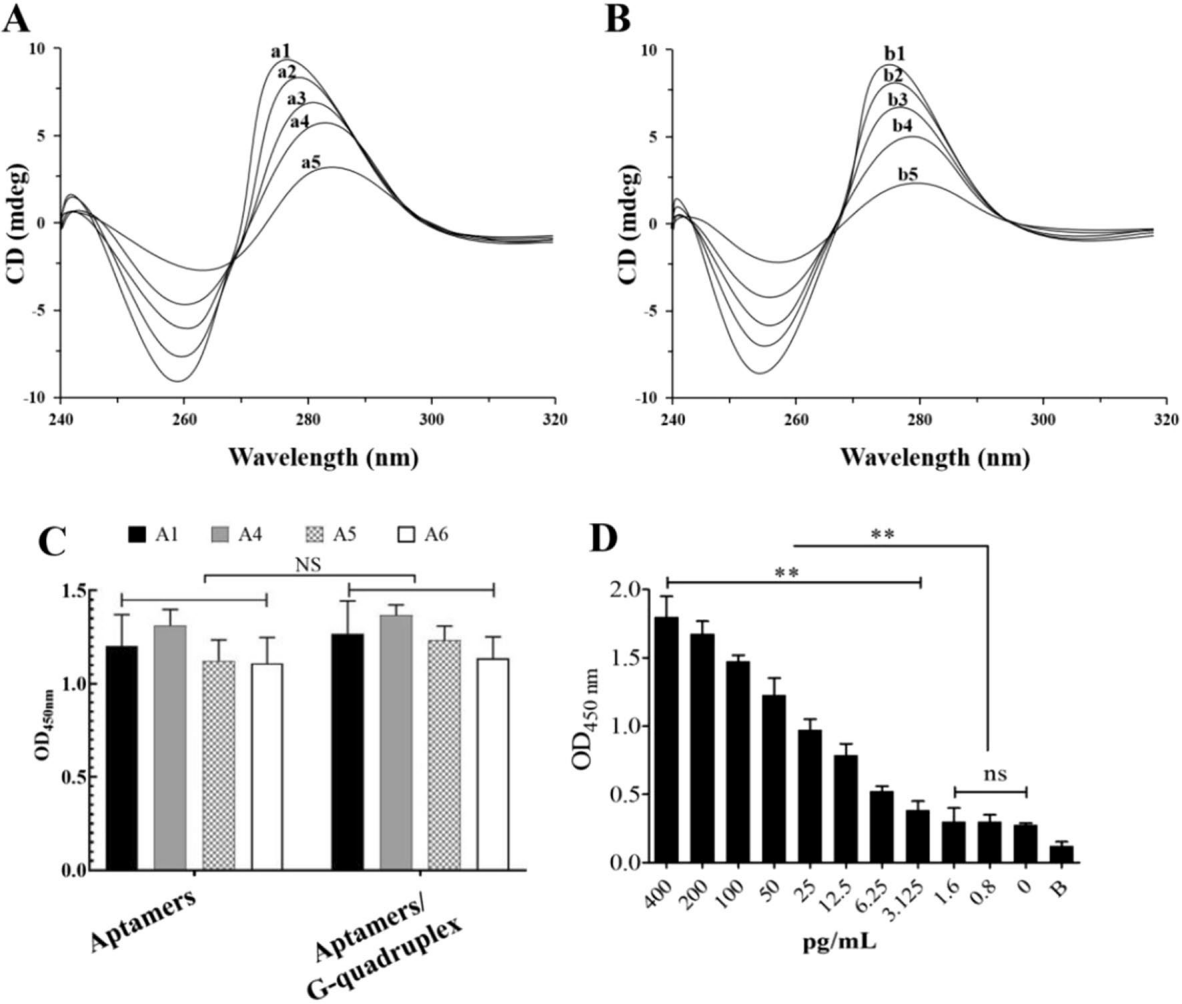
Structure identification of AGSCS and the performance of AGSCS for detection of (1 → 3)-β-D-glucans. The structure patterns of aptamers and aptamers containing G tetramer were identified by circular dichroism. **A** Common aptamers, and a5-a1 represents 0.1 mol, 0.2 mol, 0.4 mol, 0.8 mol and 1 mol. **B** Aptamer (including G tetramer), b5-b1 represents 0.1 mol, 0.2 mol, 0.4 mol, 0.8 mol and 1 mol. **C** The affinity strength of original aptamers and aptamers with G-quadruplex to (1 → 3)-β-D-glucans were assayed by enzyme-linked oligonucleotide assay. NS, non-statistical significance. **D** We evaluated the detection ability of the AGSCS for different quantities of (1 → 3)-β-D-glucans ranging from 400 to 0.8 pg/mL. All data are shown as the means ± standard deviation, and experiments were performed in triplicates

The structure patterns of common aptamers and aptamers containing G tetramer were identified by circular dichroism. The results showed that the structure of aptamers changed little after the addition of G tetramer, indicating that the addition of G tetramer would not affect the conformation of aptamers (Fig. 6A and B). To evaluate effects of G-quadruplex on binding affinity of aptamers and (1 → 3)-β-D-glucans, aptamers with G-quadruplex were added separately to polystyrene microwells coated with (1 → 3)-β-D-glucans and the effect of G-quadruplex on binding affinity of the aptamers and (1 → 3)-β-D-glucans was evaluated by ELONA. As shown in Fig. 6C, compared with original aptamers, the binding affinity of the four aptamers was not influenced by the introduced G-quadruplex fragment. The difference in binding affinity observed was statistically nonsignificant.

Next, we assessed the detection performance of the AGSCS to different concentrations of (1 → 3)-β-D-glucans. Varying concentrations of (1 → 3)-β-D-glucans were added to each well simultaneously adding the aptamers with G-quadruplex and hemin to detect the color change. Our results suggest that the limit of detection value was around 3.125 pg/mL, as observed by the naked eye (Fig. 6D). There was a good linear correlation in the range of 1.6 pg/mL to 400 pg/mL using a microplate reader-based detection (Fig. 6D). The limit of detection (1.6 pg/mL) met the requirement of (1,3)-β-D-glucan detection in IFI diagnosis.

### Detection of (1 → 3)-β-D-glucans in human serum samples using AGSCS

**Fig. 7:**
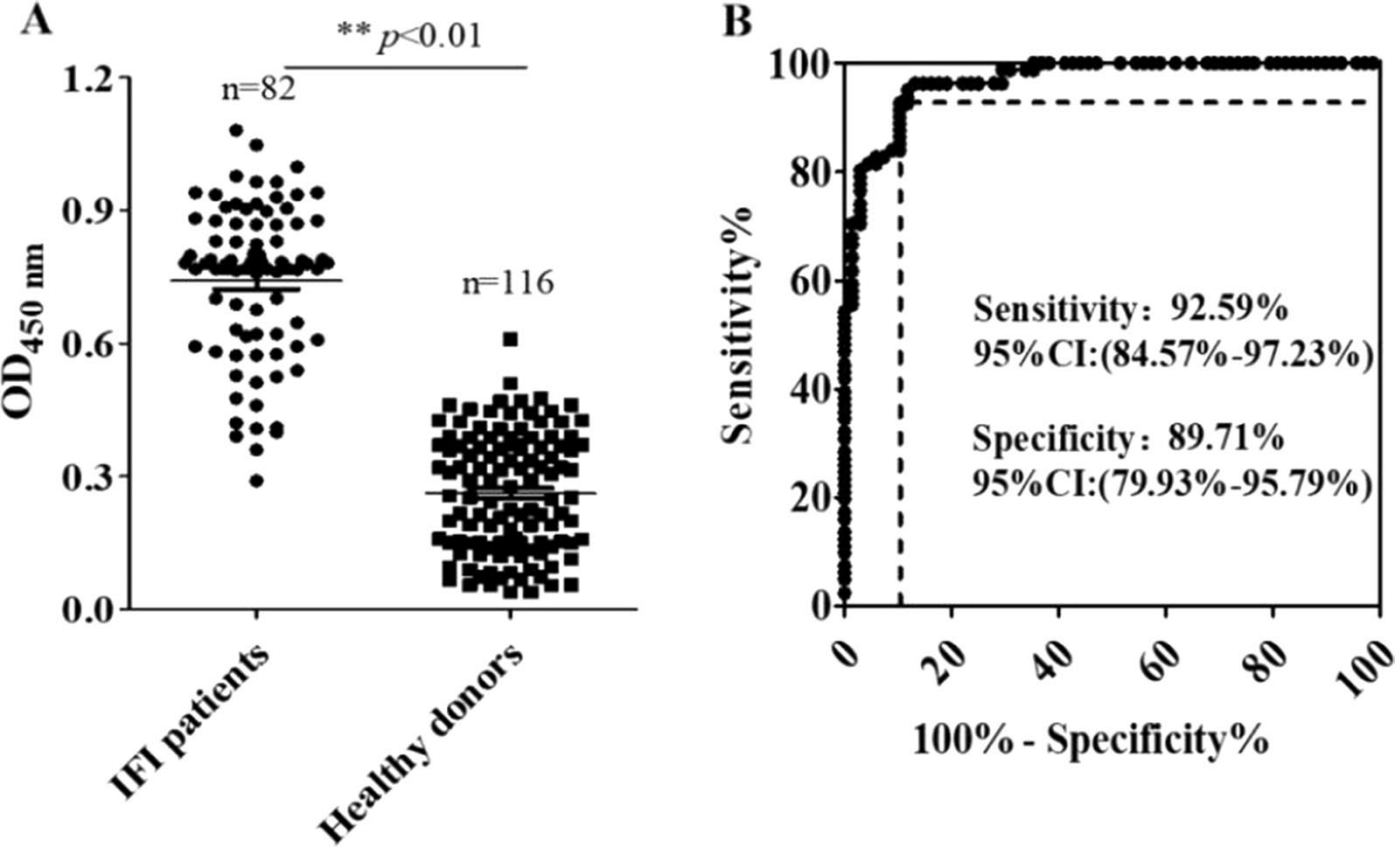
The quantification of (1 → 3)-β-D-glucans in 198 human serum specimens using aptamer G-quadruplex/hemin self-assembling color system (AGSCS). **A** A total of 198 serum samples were pipetted into 96-well microplates. Aptamers with G-quadruplex were added together with hemin and tetramethylbenzidine/H_2_O_2._ H_2_SO_4_ was subsequently added to stop the reaction of the AGSCS, and a microplate reader was used to measure the absorbance at 450 nm. All data are shown as means ± standard deviation, and experiments were performed in triplicates. **B** Receiver operating characteristics analysis of the sensitivity and specificity of AGSCS. The area under the curve was 0.9122

The sensitivity and specificity of diagnostic tests are considered vital for the proper diagnosis of diseases. Thus, in this work, 198 serum samples (82 patients with IFI and 116 healthy individuals) were used for evaluating the sensitivity and specificity of AGSCS for IFI diagnosis. As shown in Fig. 7A, patients with IFI had a significantly higher OD value compared to healthy donors. The sensitivity and specificity of AGSCS for IFI diagnosis was assessed by receiver operating characteristic (ROC) analysis. The results demonstrated the sensitivity and specificity of the assay to be 92.59% (95% confidence interval [CI]: 84.57%–97.23%) and 89.71% (95% CI: 79.93%–95.79%) respectively; while the area under the ROC curve being 0.9122 (95% CI: 0.8879–0.9451). The cut-off value was 0.4558, which was used to determine whether patients had IFI or not (Fig. 7B). These results showed that AGSCS could clearly distinguish patients with IFI from individuals without IFI.

### Detection of (1 → 3)-β-D-glucans in vivo with AGSCS

**Fig. 8:**
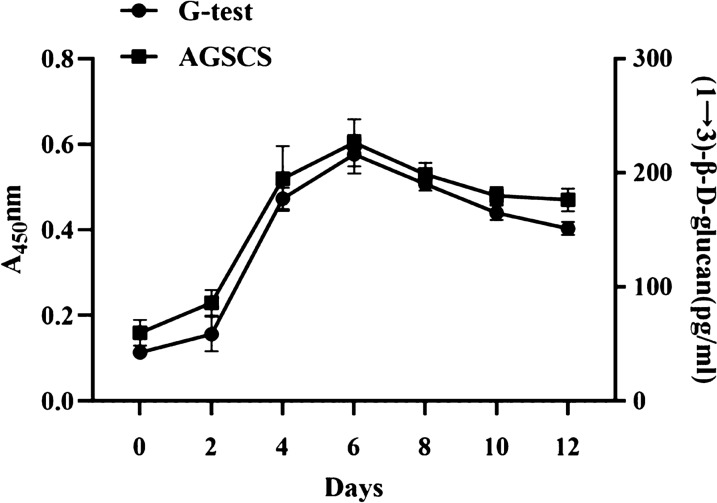
The aptamer G-quadruplex/hemin self-assembled color rendering system (AGSCS) is used to measure in vivo (1 → 3)-β-D-glucan levels. A model of *Candida albicans* infection was established using guinea pigs. Attack guinea pigs with *Candida albicans* and detect serum (1 → 3)-β-D-glucan levels every 2 days with G-test and AGSCS

To further verify whether AGSCS can detect serum (1 → 3)-β-D-glucans levels in vivo, a model of Candida albicans infection was established using guinea pigs. G testing is usually used in the treatment and monitoring of patients with invasive fungal infection [31]. As shown in Fig. 8, in vivo serum (1 → 3)-β-D-glucans levels detected by G-test and AGSCS have strong consistency. Serum (1 → 3)-β-D-glucans levels slowly rise on day 2 after *Candida albicans* injection, rise sharply on days 2 to 6. But from day 6 to day 12, we observe a gradual decrease; This may be due to the fact that triamcinolone acetonide as an immunosuppressant does not successfully inhibit the immune response of guinea pigs to *Candida albicans*, resulting in live *Candida albicans* gradually being engulfed and cleared by macrophages.

The G test has been an important tool for the diagnosis of IFI in routine clinical practice. Therefore, we further evaluated the diagnostic performance of the AGSCS compared with the G test for detecting IFI. As shown in Table 2, the sensitivity and specificity of the AGSCS compared with the G test were 92.68% and 89.65%, respectively, which were higher than those of the G test (sensitivity, 86.58%; specificity, 82.75%, *P* < 0.05). The diagnostic accuracy of the AGSCS was 90.9% thereby, higher than that of the G test (accuracy, 84.34%, *P* < 0.05).

**Table 2:** Comparison of detection performance between AGSCS and G-test for 198 clinical serum samples (Enzyme micro-plate reader)

Definite diagnosis	AGSCS		Total	G-test		Total
	Positive	Negative		Positive	Negative	
Positive	76	6	82	71	11	82
Negative	12	104	116	20	96	116
Total	88	110	198	91	107	198

The 198 serum sample from human were detected using AGSCS and G-test. The sensitivity of AGSCS and G-test were 92.68% and 86.58%, the specificity of both were 89.65% and 82.75%, respectively. The accuracy of AGSCS and G-test were 90.9% and 84.34% respectively. The AGSCS showed better detectability for patients with IFI than G-test (^2^ = 3.936, p = 0.0473).

Given our research purpose, the results of the second round of AGSCS-based detection were read by the naked eye. Here the sensitivity and specificity were 79.26% and 87.06%, respectively, and the test accuracy was 83.83%. (Table 3) These results indicated that the developed system could be effectively used for detecting (1 → 3)-β-D-glucans for IFI diagnosis.

**Table 3:** Detection performance of AGSCS for 198 clinical serum samples via naked eye observation

Definite diagnosis	AGSCS		Total
	Positive	Negative	
Positive	65	17	82
Negative	15	101	116
Total	88	110	198

The 198 serum sample from human were detected using AGSCS via naked eye observation. The sensitivity and specificity of AGSCS was 79.26% and 87.06%, and the accuracy of AGSCS was 83.83%.

## Discussion

IFI are relatively often encountered in critical care medicine and hematological patients and are associated with relatively high mortality rates [32–34]. Early diagnosis of IFI is key to reducing mortality; however, diagnosis remains a challenge. As the gold standard for IFI diagnosis, tissue culture yields positive results in only about 50% of the cases [35]. Moreover, it is difficult to extract tissue from deep anatomical sites for microbiological culture and histopathological diagnosis in critically sick patients, and histopathological examination being invasive has limited applicability in clinical practice [36]. Therefore, it is crucial to develop new diagnostic methods in medical mycology.

Glucans exist in most fungal cell walls and consist of > 60% of dry weight, among which (1 → 3)-β-D-glucans account for approximately 84% of the components in the fungal wall [25, 26]. Previous studies have suggested that all fungal cell walls except *Cryptococcus* and zygomycetes contain (1 → 3)-β-D-glucans, while viruses and other microorganisms and cell components from humans and animals do not [37]. The amount of (1 → 3)-β-D-glucans in serum appears to be very low in patients with no fungal infection, while it may increase dramatically in patients with IFI [38]. Hence, the detection of (1 → 3)-β-D-glucans in the blood can signify IFI, with the few exceptions mentioned earlier.

In this study, (1 → 3)-β-D-glucans were isolated from *C. albicans* successfully and the water-soluble low level-polymeric forms ofpolymerization of (1 → 3)-β-D-glucans were obtained through in vitro digestion with glucanase. Considering advantages of aptamers over antibodies, including high target specificity and sensitivity, easy chemical modification, and low toxicity and immunogenicity [13, 14], we screened six aptamers (A1 ~ A6) against (1 → 3)-β-D-glucans with high binding specificity and affinity. The six aptamers recognized four different epitopes of (1 → 3)-β-D-glucans.

G- quadruplex/hemin, a peroxidase-like DNAzyme, can catalyze various chemical reactions and has been widely used in biosensing [39–42]. In our study, we applied the G-quadruplex DNAzyme as a catalytic unit and aptamers as bio-recognized units to develop an AGSCS to improve the sensitivity and specificity of IFI diagnosis. Our results revealed that the limit of the detection of the AGSCS was approximately 3.125 pg/mL, with a linear dynamic correlation ranging from 1.6 pg/mL to 400 pg/mL with the microplate reader detection.

The “G test” was introduced in 1995 by Seikagaku Corporation (Tokyo, Japan) as a diagnostic test for IFI [43] and basing on glucan-activation of factor G, a protease zymogen of the horseshoe crab (*Limulus polyphemus*). Previous study reported that the G test showed a high sensitivity of 80%–90% in the diagnosis of IFI [44], and the test has been widely used in China [45, 46]. We compared the diagnostic efficiency of the AGSCS and the G test for IFI in 198 clinical serum samples. We observed that the sensitivity and specificity of the AGSCS were higher than that of the G test. In correlation analysis and models of Candida albicans infection in guinea pigs, aptamer-based AGSCS was positively correlated with the G-test, which suggests that the developed system could be relevant for the diagnosis of IFI. Meanwhile, this study explored a more feasible and convenient method for IFI diagnosis, relying on results obtained merely by the naked eye, hence being independent of sophisticated laboratory equipment. Compared with Double-Aptamer Sandwich ELONA by Tang et al., AGSCS has a larger detection range (1.6 to 400 pg/mL) [29]. Therefore, we appointed two people who knew nothing about the study before the experiment and asked them to score 198 samples with colored cards. Despite our efforts to make the scoring process as smooth, clear and objective as possible, they agreed on 196 samples and disagreed on 2 samples. This might have to do with difficulties in the scoring of very weakly positive samples. Besides, there was some color background in the detection system, which might have interfered with the visual scoring. The sensitivity and specificity of the visual method of the AGSCS were 79.26% and 87.06%, respectively, and the accuracy was 83.83%. The results obtained by the naked eye suggested a lower diagnostic efficiency than when read by the microplate reader. Meanwhile, diagnostic efficiency parameters did not differ statistically between the visual AGSCS method and the G test. Nevertheless, given the processing time, costs, and convenience, this visual AGSCS method may be a highly useful tool for IFI diagnosis and is expected to be developed as a point-of-care test kit for IFI diagnosis in the future.

Altogether, in this work, a new label-free colorimetric assay for (1,3)-β-D-glucan detection was developed based on an aptamer G-quadruplex/hemin self-assembling color system. The G-quadruplex/hemin DNAzyme-catalyzed TMB-H_2_O_2_ system yielded relevant diagnostic sensitivity. The newly developed AGSCS is expected to be a convenient, economical, time-efficient, user-friendly method for future IFI diagnosis. Furthermore, the practicability of the method holds valuable potential for the development of test kits.

## Conclusion

This newly developed visual diagnostic method for detecting IFI has shown good results, and its simplicity, speed and cost-effectiveness can be further improved to be developed into a portable point-of-care test kit that facilitates early diagnosis in the population, thereby improving the survival rate of IFI patients. In addition to this, this new diagnostic tool, combined with traditional diagnostic methods, enables better management of pathology and patients.

## Supplementary Information

**Additional file 1: Supplement document 1.** The deoxynucleotide base sequence sequences list of 6 aptamers named A1 to A6.

**Additional file 2: Supplement figure 1.** Prediction of 2 ssDNA aptamer secondary structures named A1 and A3.

**Additional file 3: Supplement figure 2.** Prediction of 2 ssDNA aptamer secondary structures named A2 and A4.

**Additional file 4: Supplement figure 3.** Prediction of 2 ssDNA aptamer secondary structures named A5 and A6.

**Additional file 5: Supplement figure 4.** (1→3)-β-D-glucans from Candida albicans was identified by silver-staining.

**Additional file 6: Supplement figure 5.** (1→3)-β-D-glucans from Candida albicans was identified by PAS.

**Additional file 7: Supplement figure 6.** Purified (1→3)-β-D-glucans from Candida albicans was identified by PAS.

## Data Availability

All data generated or analyzed during this study are included in this article. Further enquiries can be directed to the corresponding author.

## Abbreviations

IFI: Invasive fungal infection
SELEX: Systematic evolution of ligands by exponential enrichment
AGSCS: Aptamer G-quadruplex/hemin self-assembling color system
ELONA: Enzyme-linked oligonucleotide assay
FNAs: Functional nucleic acids
dsDNA: Double-stranded DNA
