# Supplementary figures and images for: A novel aptamer-G-quadruplex/hemin self-assembling color system: rapid visual diagnosis of invasive fungal infections
Source: Ann Clin Microbiol Antimicrob. 2023 May 11;22:35. doi: 10.1186/s12941-023-00570-6 (PMC10176924; doi:10.1186/s12941-023-00570-6)

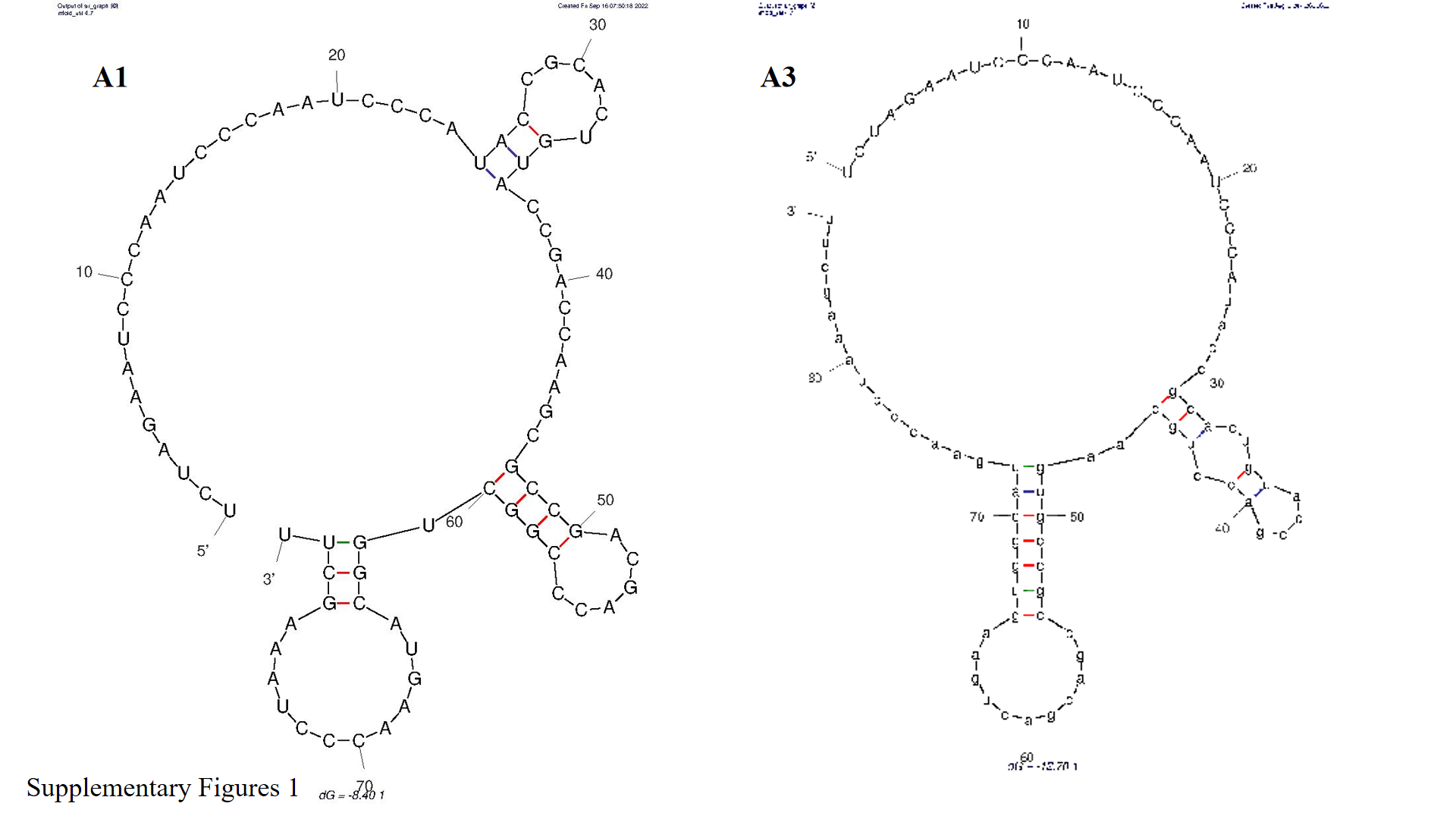

Supplement: Supplementary file 1 — Additional file 1: Supplement document 1. The deoxynucleotide base sequence sequences list of 6 aptamers named A1 to A6. [file 12941_2023_570_MOESM1_ESM.tif]

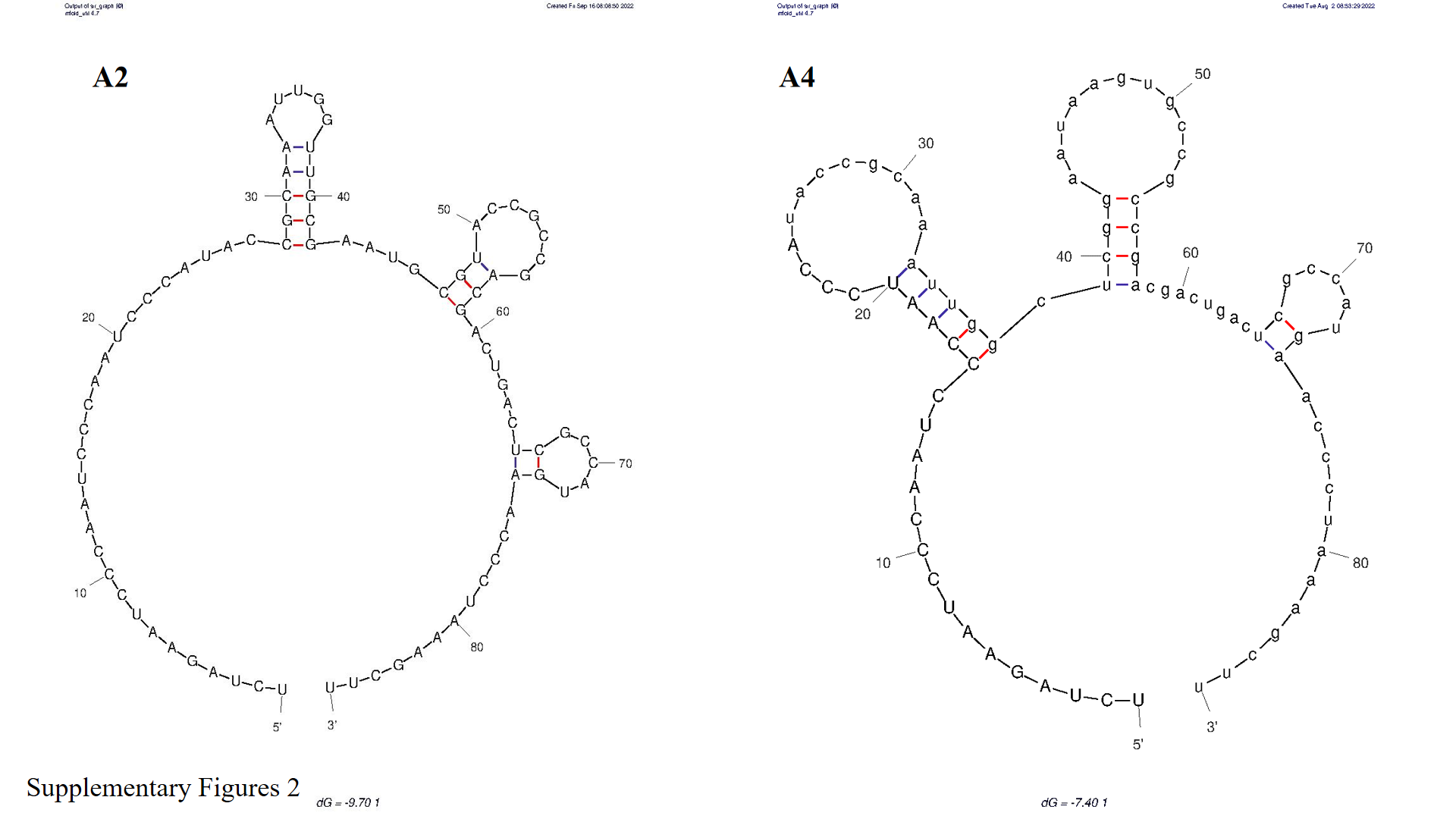

Supplement: Supplementary file 2 — Additional file 2: Supplement figure 1. Prediction of 2 ssDNA aptamer secondary structures named A1 and A3. [file 12941_2023_570_MOESM2_ESM.tif]

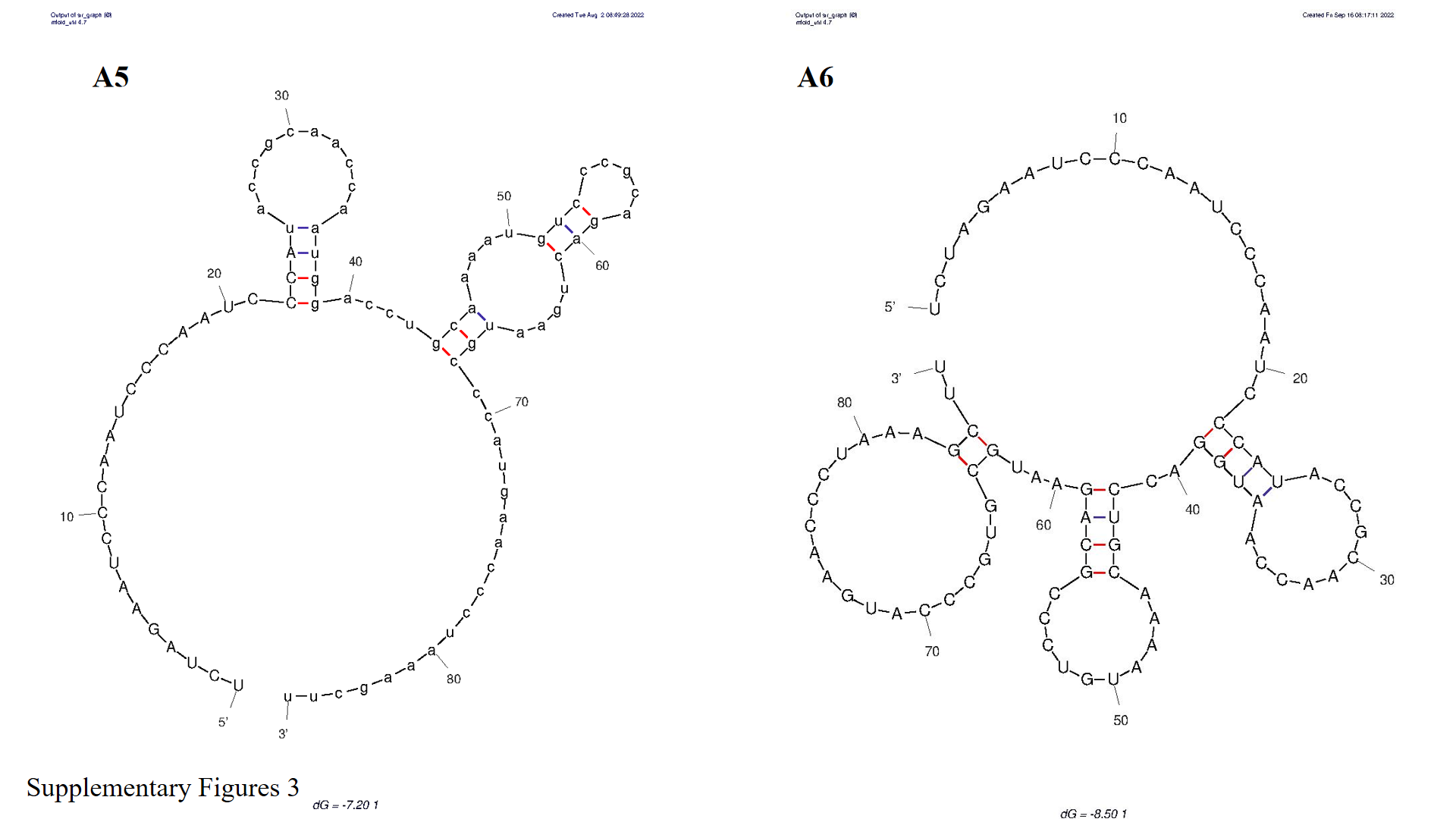

Supplement: Supplementary file 3 — Additional file 3: Supplement figure 2. Prediction of 2 ssDNA aptamer secondary structures named A2 and A4. [file 12941_2023_570_MOESM3_ESM.tif]
