# Supplementary material for: A novel aptamer-G-quadruplex/hemin self-assembling color system: rapid visual diagnosis of invasive fungal infections
Source: Ann Clin Microbiol Antimicrob. 2023 May 11;22:35. doi: 10.1186/s12941-023-00570-6 (PMC10176924; doi:10.1186/s12941-023-00570-6)
Supplement: Supplementary file 7 — Additional file 7: Supplement figure 6. Purified (1→3)-β-D-glucans from Candida albicans was identified by PAS. [file 12941_2023_570_MOESM7_ESM.docx]

**A1:**TCTAGAATCCCAATCCCAATCCCATACCGCACTGTACCGACCTGCAAGTGCCGCCGACGACTGAAGTGGCATGAACCCTAAAGCTT

**A2:**TCTAGAATCCCAATCCCAATCCCATACCGCAAATTGGCTCGGAATAAGTGCCGCCGACGACTGACTCGCCATGAACCCTAAAGCTT

**A3:**TCTAGAATCCCAATCCCAATCCCATACCGCACTGTACCGACCAAGCGCCGACGACCCGGCTGGCATGAACCCTAAAGCTT

**A4:**TCTAGAATCCCAATCCCAATCCCATACCGCAAATTGGTTGCGAATGCGTACCGCCGACGACTGACTCGCCATGAACCCTAAAGCTT

**A5:**TCTAGAATCCCAATCCCAATCCCATACCGCAACCAATGGACCTGCAAAATGTCCCGCAGACTGAATGCCCATGAACCCTAAAGCTT

**A6:**TCTAGAATCCCAATCCCAATCCCATACCGCAACCAATGGACCTGCAAAATGTCCCGCAGAATGCGTGCCCATGAACCCTAAAGCTT
